# Analysis of factors affecting intraoperative conversion from thoracoscopic radical resection of lung cancer to thoracotomy and intraoperative management experience

**DOI:** 10.12669/pjms.39.5.7422

**Published:** 2023

**Authors:** Zhen Zhang, Yuefeng Zhang, Jian Zhang, Peng Su

**Affiliations:** 1 Zhen Zhang, The Fifth Department of Thoracic Surgery, The Fourth Hospital of Hebei Medical University, Shijiazhuang 050011, Hebei, China; 2 Yuefeng Zhang, The Fifth Department of Thoracic Surgery, The Fourth Hospital of Hebei Medical University, Shijiazhuang 050011, Hebei, China; 3 Jian Zhang, The Department of Radiotherapy, The Fourth Hospital of Hebei Medical University, Shijiazhuang 050011, Hebei, China; 4 Peng Su, The Fifth Department of Thoracic Surgery, The Fourth Hospital of Hebei Medical University, Shijiazhuang 050011, Hebei, China

**Keywords:** Lung cancer, Video-assisted, Thoracoscopic surgery, Risk factor

## Abstract

**Objective:**

To explore the factors affecting the intraoperative conversion of video-assisted thoracoscopic surgery (VATS) to thoracotomy in patients with lung cancer.

**Methods:**

The clinical data of 80 patients with lung cancer in The Fourth Hospital of Hebei Medical University from May 2019 to December 2021 were retrospectively analyzed. The patients who were treated with VATS alone were included into thoracoscopy group (n= 40), and those who were intraoperatively converted from VATS to thoracotomy were included into conversion group (n= 40). The medical record data were collected, the influencing factors of intraoperative conversion from VATS to thoracotomy were analyzed, and the surgical indexes and postoperative complications were compared between the two groups.

**Results:**

Multivariate regression model showed that tumor in the upper lobe, central lung cancer, history of pulmonary tuberculosis, pleural adhesion ≥ Grade-4 and maximum tumor diameter ≥ 35 mm were risk factors for patients with lung cancer undergoing conversion from VATS to thoracotomy (*p<* 0.05). In the conversion group, the surgical duration and hospital stay were longer, the intraoperative bleeding volume and thoracic drainage volume were larger, and the total incidence of postoperative complications was higher than those in the thoracoscopy group (*p<* 0.05).

**Conclusion:**

Conversion from VATS to thoracotomy may increase the risk of complications in patients with lung cancer. Tumor in the upper lobe, central lung cancer, history of pulmonary tuberculosis, high degree of pleural adhesion and large tumor diameter are risk factors for conversion from VATS to thoracotomy.

## INTRODUCTION

Video-assisted thoracoscopic surgery (VATS) is an advanced minimally invasive diagnosis and treatment technology born with the development of imaging and minimally invasive surgery, which effectively makes up for the shortcomings of traditional thoracotomy, such as large trauma, strong stress and slow recovery. At present, it is one of the main clinical methods for the treatment of lung cancer.^1^,^2^ Although VATS has obvious advantages in the treatment of lung cancer, its biggest disadvantages compared with thoracotomy lie in unclear operating vision and limited operating space. Consequently, the clinical cases of intraoperative conversion from VATS to thoracotomy in patients with lung cancer are also common, with an incidence of about 2%~20%.^3^,^4^

Intraoperative conversion from VATS to thoracotomy is seem to cause damage to the patient’s lung and surrounding tissues due to excessive turnover and traction, and will increase the risk of intraoperative bleeding, thus prolonging the surgical duration and affecting the postoperative recovery.^5^,^6^ Analyzing and avoiding the relevant factors of intraoperative conversion from VATS to thoracotomy is an effective way to reduce its incidence. On this basis, this study analyzed the relevant factors affecting the intraoperative conversion from VATS to thoracotomy in patients with lung cancer, and discussed the experience in intraoperative management.

## METHODS

The clinical data of 80 patients with lung cancer receiving surgical treatment in The Fourth Hospital of Hebei Medical University from May 2019 to December 2021 were retrospectively analyzed. According to different surgical programs, the patients who were treated with VATS alone were included into thoracoscopy group (n = 40), and those who were intraoperatively converted from VATS to thoracotomy were included into conversion group (n= 40). All patients underwent whole-course treatment in our hospital. The study was approved by the Institutional Ethics Committee of The Fourth Hospital of Hebei Medical University (No.:2021KY279; date: June 17, 2021), and written informed consent was obtained from all participants.

### Inclusion criteria:

- Meeting the diagnostic criteria for lung cancer in the Chinese Medical Association Guidelines for Clinical Diagnosis and Treatment of Lung Cancer (Edition 2019)^7^;
- Meeting the surgical indications of VATS for lung cancer^7^;
- Lung HRCT and chest X-ray with complete and clear reports;
- Receiving VATS and thoracotomy in our hospital;
- Being informed of the surgery.

### Exclusion criteria:

- Complicated asthma, pulmonary edema and other respiratory diseases;
- Acute and chronic infection of any part;
- Severe injury of organ function and circulatory dysfunction;
- Complicated acute and chronic blood diseases;
- Severe disturbance of consciousness and poor compliance.

### Surgical methods

Before surgery, all patients underwent HRCT scanning. Routine general anesthesia combined with double-lumen endotracheal intubation, and intraoperative one-lung ventilation were performed.

### VATS method

The patients were told to fast before surgery. According to the basic information of the lesions, the patients laid on their side or flatted on the platform. The markers were routinely pasted on the body surface corresponding to the lesions. The puncture point was localized using CT positioning ray. After routine disinfection of all puncture points, local anesthesia was conducted. Combined with CT positioning information, the positioning needle was placed with appropriate method, depth and angle, and the position of the positioning needle was confirmed by CT scanning. According to the location of the lesions to be removed, the operation hole and thoracoscopic observation hole were selected between the corresponding ribs for routine resection of the lesions. After satisfactory resection, the resected specimens were taken out through the operating hole, the incision was sutured routinely, the drainage tube was indwelt, and finally VATS was ended.

### Intraoperative conversion to thoracotomy

During VATS, with difficult thoracoscopic treatment or a risk of massive bleeding, the operating hole was immediately extended to the lower angle of the scapula, with the surgical scheme changed to thoracotomy. After the ribs were opened routinely, the surgical field of vision was fully exposed, the lesions were resected and lymph nodes were dissected under direct vision, and the drainage catheter was routinely indwelt, finally followed by suturing layer by layer. All operations were performed by the same group of doctors, which has been described in the text.

### Investigation method

Combined with the medical record data, smoking history^8^, age, pathological type of lung cancer, height, course of disease, history of pulmonary tuberculosis, body weight, history of underlying diseases, tumor location, tumor diameter, ratio of FEV_1_ to forced vital capacity (FEV1/FVC), degree of pleural adhesion^9^, forced expiratory volume in first second (FEV_1_) and anatomical location of lung cancer were collected.

### Observation indexes:

- Age, gender, course of disease, body mass index (BMI), history of underlying diseases, FEV_1_/FVC and FEV_1_ were compared between the two groups.
- The correlations of conversion from VATS to thoracotomy with smoking history, age, pathological type of lung cancer, BMI, course of disease, history of pulmonary tuberculosis, history of underlying diseases, tumor location, tumor diameter, FEV_1_/FVC, degree of pleural adhesion, FEV_1_ and anatomical location of lung cancer were analyzed.
- Combined with the results of clinical data comparison and univariate analysis, a logistic regression model was established for multivariate regression analysis of VATS conversion to thoracotomy.
- The surgical duration, intraoperative bleeding volume (volume method + weighing method), indwelling time of drainage tube, hospital stay, thoracic drainage volume and postoperative complications were compared between the two groups. The adverse reactions of the two groups of patients within one month after medication were recorded.

### Statistical Analysis

The medical record data were imported and sorted out with Excel 2019, and statistically analyzed using SPSS 22.0. The enumeration data were analyzed by the *x^2^* test, and the measurement data by the t-test. The relevant influencing factors of VATS conversion to thoracotomy were screened using multivariate logistic regression analysis (screening criteria, *p<* 0.20). *P<* 0.05 was considered statistically significant.

## RESULTS

Age, BMI, history of underlying diseases, gender, FEV_1_, course of disease and FEV_1_/FVC showed no statistically significant differences between the two groups (*p>* 0.05) (Table-I). In history of pulmonary tuberculosis, tumor location, maximum tumor diameter, degree of pleural adhesion and anatomical location between the two groups (*p<* 0.05) (Table-II).

**Table-I:** Comparison of baseline data between two groups.

Index		Conversion group (n = 40)	Thoracoscopy group (n = 40)	x^2^/t	P
Age (year, *x̅* ± s)		62.27±3.51	62.35±3.62	1.110	0.270
BMI (kg/m^2^, *x̅* ± s)		22.16±3.78	20.43±3.52	0.351	0.727
Course of disease (month, *x̅* ± s)		6.65±0.57	6.79±0.46	0.163	0.871
FEV1 (L, ±s)		1.75±0.35	1.72±0.42	0.348	0.729
FEV1/FVC (%, *x̅* ±s)		76.73±4.52	76.85±4.67	0.117	0.907
Gender [n, (%)]	Male	22(55.00)	23(57.50)	0.051	0.822
	Female	18(45.00)	17(42.50)		
History of underlying diseases [n, (%)]	Hyperlipidemia	11(27.50)	12(30.00)	0.061	0.805
	Diabetes	13(32.50)	10(25.00)	0.549	0.459
	Hypertension	5(12.50)	3(7.50)	0.139	0.709

**Table-II:** Univariate analysis of conversion from VATS to thoracotomy [n, (%)]

Index		Conversion group (n = 40)	Thoracoscopy group (n = 40)	x^2^	P
Age (year)	< 65	17(42.50)	14(35.00)	0.474	0.491
	≥ 65	23(57.50)	26(65.00)		
BMI (kg/m^2^)	< 28	34(85.00)	31(77.50)	0.739	0.390
	≥ 28	6(15.00)	9(22.50)		
Smoking history	Yes	24(60.00)	22(55.00)	0.205	0.651
	No	16(40.00)	18(45.00)		
Pathological type	Adenocarcinoma	25(62.50)	27(67.50)	0.320	0.852
	Squamous cell carcinoma	12(30.00)	11(27.50)		
	Others	3(7.50)	2(5.00)		
History of pulmonary tuberculosis	Yes	25(62.50)	13(32.50)	7.218	0.007
	No	15(37.50)	27(67.50)		
Maximum tumor diameter(mm)	< 35	12(30.00)	27(67.50)	11.257	0.001
	≥ 35	28(70.00)	13(32.50)		
Degree of pleural adhesion (grade)	< 4	19(47.50)	33(82.50)	10.769	0.001
	≥ 4	21(52.50)	7(17.50)		
Tumor location	Upper lobe	22(55.00)	8(20.00)	10.453	0.001
	Other parts	18(45.00)	32(80.00)		
FEV_1_ (L)	< 1.70	21(52.50)	18(45.00)	0.450	0.502
	≥ 1.70	19(47.50)	22(55.00)		
FEV_1_/FVC (%)	< 75	17(42.50)	19(47.50)	0.202	0.653
	≥ 75	23(57.50)	21(52.50)		
Anatomical location	Central	25(62.50)	11(27.50)	9.899	0.002
	Peripheral	15(37.50)	29(72.50)		

Multivariate regression model showed that tumor in the upper lobe (95%CI: 0.513~0.825; OR: 1.695), central lung cancer (95%CI: 0.579~0.861; OR: 1.733), history of pulmonary tuberculosis (95%CI: 0.435~0.895; OR: 2.835), pleural adhesion ≥ grade 4 (95%CI: 0.605~0.913, OR: 2.841) and maximum tumor diameter ≥ 35 mm (95%CI: 0.769~0.925; OR: 3.023) were risk factors for patients with lung cancer undergoing conversion from VATS to thoracotomy (*p<* 0.05) (Table-III).

**Table-III:** Multivariate regression analysis of conversion from VATS to thoracotomy.

Factor	β	Wald x^2^	SE	OR	P	95%CI
Tumor location (upper lobe)	0.220	2.239	0.150	1.695	0.001	0.513~0.825
Central lung cancer	0.311	3.169	0.145	1.733	0.001	0.579~0.861
History of pulmonary tuberculosis	1.353	12.619	0.110	2.835	< 0.001	0.435~0.895
Degree of pleural adhesion ≥grade 4	1.580	7.623	0.211	2.841	< 0.001	0.605~0.913
Maximum tumor diameter ≥ 35 mm	1.283	5.795	0.361	3.023	< 0.001	0.769~0.925

In the conversion group, the surgical duration and hospital stay were longer, the intraoperative bleeding volume and thoracic drainage volume were larger, and the total incidence of postoperative complications was higher than those in the thoracoscopy group (*p<* 0.05, Table-IV).

**Table-IV:** Comparison of surgical indexes and complications between two groups [n, (%)].

Index		Conversion group (n = 40)	Thoracoscopy group (n = 40)	x^2^/t	P
Surgical duration (min, *x̅* ± s)		203.53±37.59	158.76±31.62	5.764	0.000
Indwelling time of drainage tube (d, *x̅* ± s)		7.13±2.20	6.76±2.05	0.778	0.439
Hospital stay (d, *x̅* ± s)		14.53±3.11	8.25±2.50	9.954	0.000
Intraoperative bleeding volume (ml, *x̅* ± s)		976.59±68.86	357.45±43.62	48.039	0.000
Thoracic drainage volume (ml, *x̅* ± s)		1013.59±159.36	895.85±102.57	3.929	0.000
Postoperative complications	Pulmonary atelectasis	3(7.50)	1(2.50)		
	Persistent pulmonary air leakage	2(5.00)	0		
	Respiratory failure	1(2.50)	0		
	Pulmonary infection	9(22.50)	3(7.50)		
	Subcutaneous emphysema	0	2(5.00)		
	Total incidence	15(37.50)	6(15.00)	5.230	0.022

## DISCUSSION

In the present study, the results showed that tumor in the upper lobe (OR: 1.695), central lung cancer (OR: 1.733), history of pulmonary tuberculosis (OR: 2.835), pleural adhesion ≥ Grade-4 (OR: 2.841) and maximum tumor diameter ≥ 35 mm (OR: 3.023) were risk factors for patients with lung cancer undergoing conversion from VATS to thoracotomy, which is consistent with the results of Liu Y et al.^10^

The findings indicate that clinical intervention can be focused on lung cancer patients with the above risk factors, so as to improve the rationality and scientificity of the surgical scheme and reduce the incidence of conversion from VATS to thoracotomy. In addition, it was also found that the intraoperative bleeding volume was larger, the incidence of postoperative complications was higher, and the length of hospital stay was longer in the conversion group than those in the thoracoscopy group, which is similar to the study of Bongiolatti S et al.^11^, suggesting that the conversion from VATS to thoracotomy in patients with lung cancer can reduce the quality of prognosis, increase the risk of complications and prolong the time of recovery.

Lung cancer is the malignant tumor with the highest mortality in China, accounting for about 18% of all malignant tumor-caused deaths, which has brought a heavy burden to China’s public health system.^12^,^13^ Surgical resection of lesions can effectively inhibit the progression of lung cancer and improve the five-year survival rate of patients.^14^ Since the first study on VATS was reported in the 1990s, VATS has been increasingly used after more than 30 years of development.^15^ VATS has the advantages of accurate positioning, small trauma, mild pain and rapid recovery. Because thoracoscope can clearly display the enlarged tissue images, VATS has a good surgical field of vision, which contributes to almost all thoracic surgery performed under thoracoscope.^16^ Nevertheless, VATS still has less obvious advantages than thoracotomy in surgical accuracy, surgical field and operating space.^17^

In view of the disadvantages of VATS compared with thoracotomy, some patients with lung cancer often have to temporarily change the surgical scheme to thoracotomy during VATS. The conclusion of this study suggests that thoracotomy switching from VATS can prolong the operation time of lung cancer patients and increase the risk of intraoperative bleeding and complications

Based on the long-term clinical experience and previous literature analysis^18^-^20^, the author summarizes the countermeasures for VATS conversion to thoracotomy as follows: (1) Hilar or mediastinal calcification and adhesion, lymph node enlargement and pleural adhesion can increase the operational difficulty of patients with lung cancer undergoing VATS, as well as the risk of massive bleeding. The risk is higher in patients with tumor in the upper lobe, central lung cancer, history of pulmonary tuberculosis, high degree of pleural adhesion and larger tumor diameter. Therefore, lung cancer patients with such conditions are the main population undergoing VATS converted to thoracotomy.^18^ (2) In patients with lung cancer undergoing VATS, if the risk that may lead to a difficulty in thoracoscopic treatment or massive bleeding is found, they should be timely converted to thoracotomy. Additionally, unnecessary turnover and traction during thoracotomy should be avoided to protect the patients’ lung tissue.^19^ (3) The conversion of VATS to thoracotomy will increase the risk of complications. Consequently, we should actively monitor the postoperative signs and complications, so as to prevent and control complications such as pulmonary infection and pulmonary atelectasis.^20^ The findings of this study adds to the clinical data on the risk factors associated with conversion from VATS to thoracotomy.

### Limitations

This was a retrospective descriptive study, with limited clinical data available and limited persuasive conclusions. Further intervention trials are needed in the future to confirm these results.

## CONCLUSION

Conversion from VATS to thoracotomy may prolong the surgical duration, as well as increase the risk of intraoperative bleeding and complications in patients with lung cancer. Tumor in the upper lobe, central lung cancer, history of pulmonary tuberculosis, high degree of pleural adhesion and large tumor diameter are the risk factors for conversion from VATS to thoracotomy.

### Authors’ Contributions:

**ZZ** and **PS:** Designed this study, prepared this manuscript, are responsible and accountable for the accuracy and integrity of the work. **YZ:** Collected and analyzed clinical data. **JZ:** Participated in acquisition, analysis, or interpretation of data and draft the manuscript.
